# Banana Leaf Surface’s Janus Wettability Transition from the Wenzel State to Cassie–Baxter State and the Underlying Mechanism

**DOI:** 10.3390/ma15030917

**Published:** 2022-01-25

**Authors:** Yinlong Jiang, Zhou Yang, Tingting Jiang, Dongying Shen, Jieli Duan

**Affiliations:** 1 College of Engineering, South China Agricultural University, Guangzhou 510642, China; jiangyun@stu.scau.edu.cn (Y.J.); 2385390222@stu.scau.edu.cn (T.J.); 20203163054@stu.scau.edu.cn (D.S.); 2 Guangdong Laboratory for Lingnan Modern Agriculture, Guangzhou 510642, China; 3 Guangdong Provincial Key Laboratory of Conservation and Precision Utilization of Characteristic Agricultural Resources in Mountainous Areas, Jiaying University, Meizhou 514015, China

**Keywords:** microstructure, dynamic wetting behavior, wetting model, biomaterials

## Abstract

Janus wettability plays an important role in certain special occasions. In this study, field emission scanning electron microscopy (FESEM) was used to observe the surface microstructure of banana leaves, the static wettability of the banana leaf surface was tested, and the dynamic response of water droplets falling at different heights and hitting on the adaxial and abaxial sides was studied. The study found that the nanopillars on the adaxial and abaxial sides of the banana leaf were different in shape. The nanopillars on the adaxial side were cone-shaped with large gaps, showing hydrophilicity (Wenzel state), and the heads of the nanopillars on the abaxial side were smooth and spherical with small gaps, showing weak hydrophobicity (Cassie–Baxter state). Banana leaves show Janus wettability, and the banana leaf surface has high adhesion properties. During the dynamic impact test, the adaxial and abaxial sides of the banana leaves showed different dynamic responses, and the wettability of the adaxial side of the banana leaves was always stronger than the abaxial side. Based on the structural parameters of nanopillars on the surface of the banana leaf and the classical wetting theory model, an ideal geometric model around a single nanopillar on both sides of the banana leaf was established. The results show that the established model has high accuracy and can reflect the experimental results effectively. When the apparent contact angle was 76.17°, and the intrinsic contact angle was 81.17° on the adaxial side of the banana leaf, steady hydrophilicity was shown. The abaxial side was similar. The underlying mechanism of Janus wettability on the banana leaf surface was elucidated. This study provides an important reference for the preparation of Janus wettability bionic surfaces and the efficient and high-quality management of banana orchards.

## 1. Introduction

With the evolution of biology, a specific structure has been formed on the surface of animals and plants to adapt to the living environment, which has also become one of the hotspots of biologically efficient production management and bionics research. For example, the surface of rice leaves and butterfly wings show anisotropy [1,2]; the surfaces of prickly pear cacti (*Opuntia*) show age-dependent wetting properties [3]; the surface of lotus leaves show super-hydrophobicity [4], and the surfaces of cicada wings show gradient wettability along the nanopillars, which achieves an efficient fog harvest while ensuring a clean and light surface [5,6]. The “Van der Waals force” generated between the large number of fine hairs on the soles of the gecko’s feet and the molecules on the surface of the object is accumulated to form a high adhesion force, which enables it to travel freely on the wall [2]. In addition, the researchers also conducted research on a large number of animal and plant surfaces, biological by-products, etc., and carried out the exploration of bionic material preparation and efficient production and management of biology [7,8,9].

Janus wettability was an important wetting property discovered in recent years, and extensive research has been carried out [10,11,12]. Inspired by the Janus wettability, researchers have carried out research on the wettability of water and oil in the air and the first preparation of Janus membranes with a directional water-transport property [13]. Additionally, they have prepared a hydrophobic copper mesh and cotton layer with higher fog-harvesting efficiency than a single material [14]. The hydrophilic fabric was prepared by one-side electro-spraying technology, and the water-transport property on both sides was qualitatively studied [15]. In the same way, electro-spun membranes have been prepared, which have higher oil-water separation efficiency than single-layer superhydrophobic-oleophilic membranes. Significant progress has been made in oil-water separation and fog harvest [16]. Researchers are also constantly exploring natural surfaces with Janus wettability, providing references for the preparation of various new microstructures and bio-efficient production and management.

As an important biological template, banana leaves have significant properties, such as self-cleaning [17] and enhancing dropwise condensation [18]. Researchers have prepared a variety of bionic materials and equipment from bionic banana leaves [19]. In previous work, it has been revealed that the surface of banana leaves exhibits Janus wettability [20], which provides an important reference for the preparation of biomimetic materials and efficient production management of banana orchards. Literature-review studies found that, at present, although the surface-wetting theory and the relationship between the micro nanopillars and wettability coupling launched a lot of research, there are few reports on the elaboration of the microscale mechanism, especially the construction of an ideal wetting geometric model. This is of great significance for the preparation of biomimetic materials and the selection of wetting gradients. Therefore, we carry out relevant research in this paper.

The main work of this research is to reveal the wetting mechanism of the banana leaf surface, study the effect of water droplets on the dynamic wettability of the banana leaf surface, and clarify the Janus wettability transition from the Wenzel state to the Cassie-Baxter state and its underlying mechanism. This is of great significance for the bionic application on the surface of banana leaves and the efficient and high-quality production and management of banana orchards, especially for the guidance of pesticide spraying.

## 2. Materials and Methods

### 2.1. Starting Materials

Fresh banana leaves (plantain banana) that were in good health, free from pests, diseases, and mechanical damage were picked from South China Agricultural University, and the parts between the veins and edges of the banana leaves were selected as test samples. In order to ensure the cleanliness of the banana leaf surface and the accuracy of the experimental results, the test samples were taken from a banana plant aged between 7 and 9 months, and take the third healthy banana leaf from top to bottom. Deionized water was then used to ultrasonically clean the banana leaves for 60 s (KQ-600DE, Kunshan Ultrasonic Instrument Co., Ltd., Kunshan, China) to remove the physical attachments on the surface of the banana leaves.

### 2.2. Surface Characterization of Banana Leaves

Using a contact angle measuring instrument (JC2000D1, Shanghai Zhongchen Digital Technology Apparatus Co., Ltd., Shanghai, China) to measure the water contact angles (CAs) and rolling angles (RAs, the inclination angle of the banana leaf when the water droplets reach a critical state before rolling on the surface of the inclined banana leaf) of 6 μL droplets on the adaxial and abaxial sides of the banana leaves, they were measured at three different positions to obtain the average value. At the same time, the ultrasonically cleaned banana leaves were vacuum dried in a vacuum drying oven at 40 °C, then cut into 5 mm × 5 mm size, and gold sprayed through vacuum sputtering to make the sample conductive. Field emission scanning electron microscopy (FESEM; Verios 460, Thermo Fisher, Waltham, MA, USA) was used to observe the microstructure of the adaxial and abaxial sides of the banana leaves. During the FESEM imaging process, the banana leaves were first pasted on the sample holder with conductive glue, and then sprayed gold with vacuum sputtering and observed under scanning electron microscopy. The detector type was SE, the electron beam intensity was 1.00 kV, and the working distance was 4.1 mm.

### 2.3. Dynamic Impact Test

**Figure 1:**
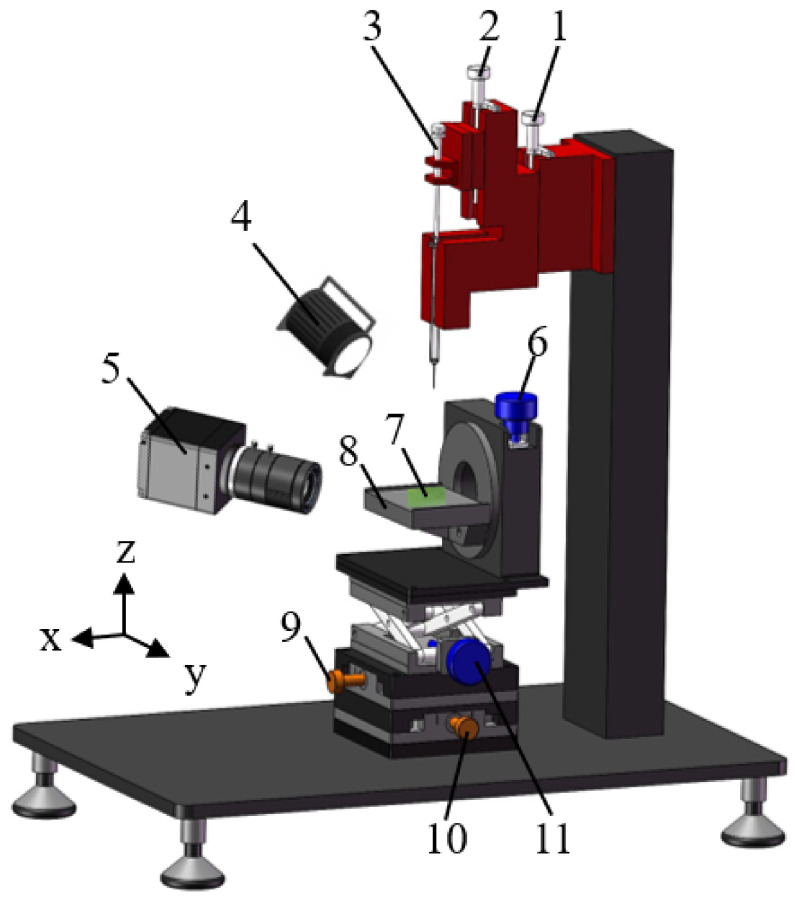
Dynamic impact test platform, 1. Height adjustment knob, 2. Droplet size adjustment knob, 3. Precision syringe, 4. Light source, 5. High-speed camera, 6. Angle adjustment knob, 7. Banana leaf sample, 8. Stage, 9. X axis adjustment knob, 10. Y axis adjustment knob, 11. Z axis adjustment knob.

The self-made dynamic impact test platform shown in Figure 1 was used for the water droplet dynamic impact tests. The test environment was a room temperature of 24 °C ± 1 °C and a humidity of 70% ± 10%. The water droplet impact test was used to evaluate the dynamic wettability of the banana leaf surface. The volume of the water droplet used for the test was *V* = 8 μL (initial diameter was about 2.48 mm), and the surface tension of water was *γ* = 0.072 N·m^−1^. Water droplets fell freely from different heights (*h*) and impacted the surface of the banana leaf sample. Under the action of gravity, an impact velocity (*v*) was obtained, and *v* can be expressed as the following equation:

(1) $$v=\sqrt{2gh}$$

where *g* is the acceleration of gravity (9.8 m·s^−2^). Water droplets dropped from heights of 20 mm, 50 mm, 100 mm, 150 mm, and 200 mm to impact the banana leaf surface, and a high-speed camera (Fastcam Mini UX50, Photron, Tokoy, Japan) was used to record the impact process of water droplets on the banana leaf surface at a speed of 2000 fps, so as to test the dynamic wettability of the water droplets on the adaxial and abaxial sides of the banana leaf.

## 3. Results and Discussion

### 3.1. Surface Characteristics of Banana Leaves

Wettability is an important index to evaluate the surface properties of materials. The water contact angles (CAs) and rolling angles (RAs) of the banana leaf surface were measured at three different positions (Tip, Middle, and Base) on the adaxial and abaxial sides. The test results of the contact angles of 6 μL water droplets on the banana leaf surface showed that the contact angle on the adaxial side was 76.08° ± 2.01°, showing hydrophilicity, and the contact angle on the abaxial side was 94.32° ± 1.04°, showing weak hydrophobicity. In addition, the adaxial and abaxial sides of the banana leaf have high adhesion, the rolling angle is bigger than 90°, and the adaxial and abaxial sides of the banana leaves show Janus wettability.

A large number of studies have shown that the surface microstructure is the main element that affects the wettability of the materials [1,6,21]. Field emission scanning electron microscopy (FESEM) was used to observe and research the microstructure of the adaxial and abaxial sides of the banana leaves. The study found that both the adaxial and abaxial sides of the banana leaf were distributed with nanoscale papillary nanopillar structures. The nanopillars on the adaxial side of the banana leaves were in a conical shape, and the gap between the two adjacent nanopillars was relatively large. The top of the nanopillars on the abaxial side of the banana leaves was a smooth spherical shape, and the gap between the two adjacent nanopillars was small. The structure parameters of the nanopillars on the adaxial and abaxial sides of the banana leaf are shown in Table 1.

**Figure 2:**
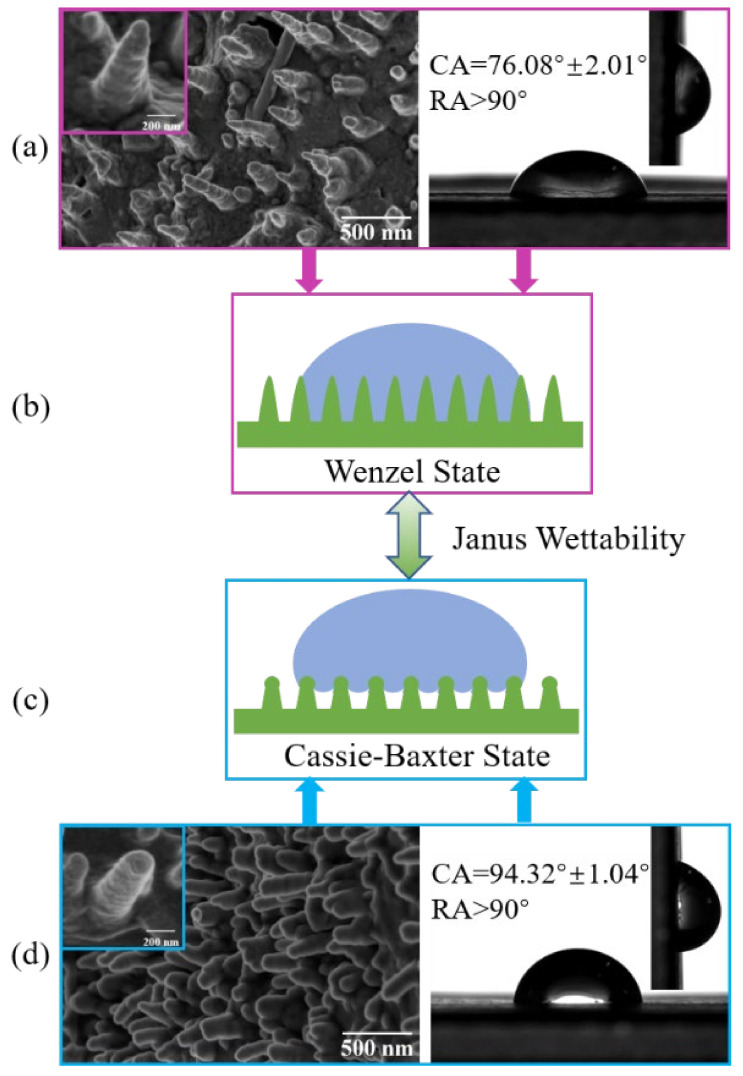
Banana leaves surface characteristics. (**a**,**b**) The adaxial side; (**c**,**d**) the abaxial side; (**a**,**d**) the nanopillar structure on the left and the contact angle and rolling angle on the right; (**b**,**c**) the wetting model.

The analysis of the test results shows that the main reason for the Janus wettability on the banana leaf surface was the difference in the structure and size of the nanopillars [20]. The nanopillars’ structure on the adaxial side of the banana leaves was sharp, the gap between adjacent nanopillars was relatively large, and water droplets were easily pierced, remaining in the gap between the adjacent nanopillar. The head of the nanopillars on the abaxial side of the banana leaf was smooth and spherical, which can effectively prevent water droplets from continuing to infiltrate the surface, forming air pockets, and showing weak hydrophobicity. Its structure and wetting model are shown in Figure 2.

From Figure 2a, it can be found that the nanopillars on the adaxial side of the banana leaf were tapered and had a large spacing. The wetting performance was hydrophilic, with a hydrophilic contact angle and a higher rolling angle. Corresponding to Figure 2d, the top of the nanopillars on the abaxial side of the banana leaf was spherical, and there is a small spacing between the nanopillars, which directly leads to the weak hydrophobicity on the abaxial side of the banana leaf. However, the reverse side of the banana leaf also has a higher rolling angle, and both sides of the banana leaf show obvious Janus wettability and adhesion. On this basis, as shown in Figure 2b,c, we combined the classical hydrophilic Wenzel equation and hydrophobic Cassie–Baxter equation to establish a geometric model of ideal wetting states on the adaxial side and abaxial of the banana leaves. In order to verify the accuracy of the model and the dynamic stability of wettability, the dynamic impact experiment was continued to reveal the underlying mechanism for Janus wettability on the banana leaf surface and to conduct a comparative study between the established model and real experiments.

### 3.2. Dynamic Wetting Behaviors on Banana Leaf Surface

**Figure 3:**
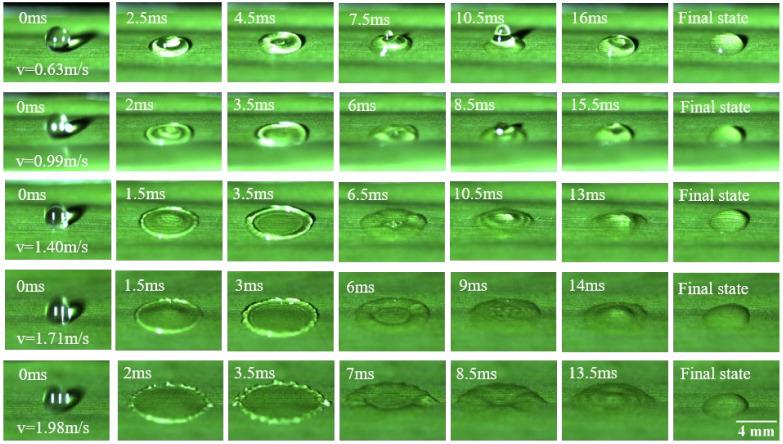
Typical snapshots of droplet impacting at abaxial side.

A dynamic impact test was an important technical method to explore the robustness and dynamic stability of banana leaf surface wetting behavior. By adjusting the drop height of the water droplets, the impact velocity was controlled to be 0.63 m·s^−1^, 0.99 m·s^−1^, 1.40 m·s^−1^, 1.71 m·s^−1^, and 1.98 m·s^−1^, respectively. Figure 3 shows the typical snapshots of the wetting behavior when water droplets hit the abaxial side of the banana leaves at different velocities. After the water droplets hit the surface of the banana leaves, they spread to the maximum in about 3.5 ms. The center of the spreading water was concave, and the edges were convex and smooth. As the impact velocity increases, wavy lines gradually appear on the edges of the droplets, and after multiple retractions and rebounds, the kinetic energy of the water droplets decays to zero, and they remain hydrophilic when they are finally stabilized on the banana leaf surface. Similarly, when water droplets hit the adaxial side of the banana leaves, they show similar performance.

**Figure 4:**
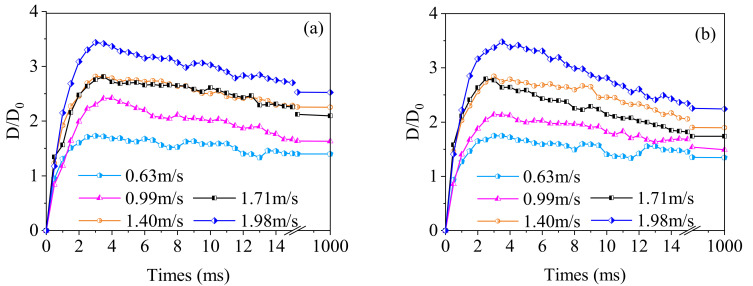
Relation curve between spreading factor with time. (**a**) Adaxial side; (**b**) abaxial side.

Furthermore, we define the initial diameter of the water droplets as *D*_0_, and the diameter of the water droplets during the spreading process as *D*. The relation curve between the water droplets spreading factor β ($\beta =D/D_{0}$) and time when the water droplets hit the surface of the banana leaves is shown in Figure 4. Obviously, the adaxial and abaxial sides of the banana leaf show the same trend. With the increase in the impact velocity, the maximum value of β gradually increases, and the change trend was the same under different impact velocities.

In Figure 4, we were surprised to find that, no matter whether on the adaxial or abaxial side of the banana leaf, when the droplet impacted the surface of the banana leaf at a velocity of 1.40 m·s^−1^, the spreading factor of the droplets had a higher stability, and the spreading factor was obviously improved in the final steady-state. When the impact velocity was low, the kinetic energy of the droplet was small. During the energy-conversion process of the spreading stage, due to the conversion of kinetic energy and internal energy, the droplet shrinks and fluctuates greatly. However, when the impact velocity is high, the larger kinetic energy makes the droplets splash during the spreading and retracting stages, thus leading to the fluctuation in the spreading factor with time.

**Figure 5:**
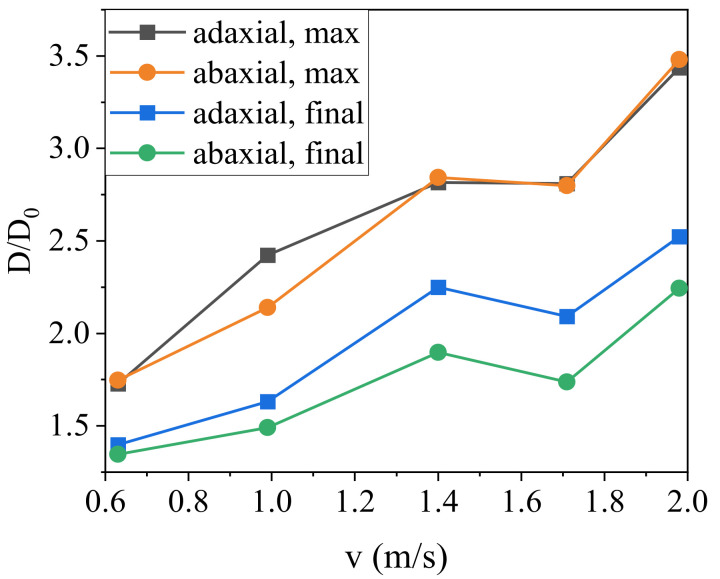
Maximum spreading factor and final spreading factor.

In order to further explore the dynamic impact behavior of water droplets on the surface of banana leaves, Figure 5 shows the maximum spreading factor and the final steady-state spreading factor when the water drop hits the surface of the banana leaf. The results show that when the droplet hits the banana leaf at a velocity of 1.40 m·s^−1^, the maximum spreading factor and the final steady-state spreading factor are relatively stable, and show a small peak. When the impact velocity is too low, there will be large fluctuations due to multiple fluctuations during the spreading phase of the droplets. When the impact velocity increases, the spreading factor will first decrease and then increase. If the velocity is too high, it may cause the droplets to break and splash. This provides an important reference for the design of pesticide spraying equipment and the adjustment of operating parameters in banana orchards.

The test results of the impact behavior of water droplets on the surface of banana leaves show that in the final steady-state, the spreading factor of the adaxial side is always bigger than that of the abaxial side, indicating that the wettability of adaxial sides is always better than the abaxial side under the dynamic impact of water droplets. The banana leaf surface shows stable Janus wettability and better robustness.

### 3.3. Mechanism Analyses on Janus Wettability

The adaxial side of banana leaves showed a hydrophilic Wenzel state, the nanopillars were completely infiltrated, and its hydrophilic state was analyzed by the Wenzel wetting mechanism. Assuming that the apparent contact angles (CAs) of the banana leaf surface are θ* and the intrinsic contact angle is θ, the relationship can be expressed by the following Wenzel equation [22]:

(2) cosθ*=rcosθ

where r is the ratio of the real contact area ($S_{a}$) of the water droplet and the adaxial side of the banana leaf surface to its projected area ($S_{p}$):

(3) $$r=\frac{S_{a}}{S_{p}}$$

**Figure 6:**
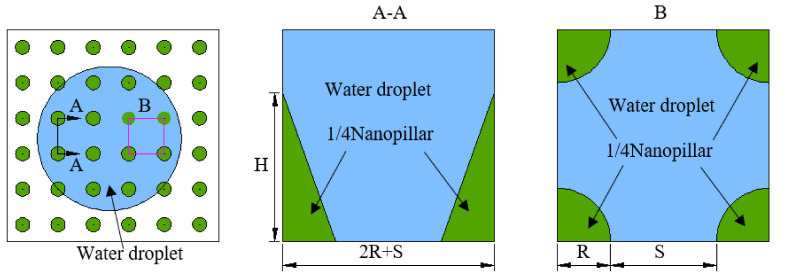
Idealized geometric model of the banana leaf’s adaxial side.

On the rough banana leaf surface, $S_{a}>S_{p}$ was always established and r was always bigger than 1. In order to calculate r, as shown in Figure 6, an idealized geometric model of a single nanopillar and the surrounding area was established according to the nanopillars parameters (Table 1) and the Wenzel wetting state model (Figure 2b) on the banana leaf’s adaxial side.

According to the contact relationship between the nanopillars and water droplets in Figure 6, $S_{a}$ and $S_{p}$ can be expressed as follows:

(4) $$S_{a}=\pi R\left(\sqrt{H^{2}+R^{2}}-R\right)+{\left(S+2R\right)}^{2}$$

(5) $$S_{P}={\left(S+2R\right)}^{2}$$

Thus, combining Equations (2)−(5), Equation (2) can be rewritten as:

(6) $$\cos\theta *=\left[1+\frac{\pi R\left(\sqrt{H^{2}+R^{2}}-R\right)}{{\left(S+2R\right)}^{2}}\right]\cos\theta$$

Substituting the nanopillars parameters *R*, *H*, and *S* of the banana leaves’ adaxial side (Table 1) with the average value of apparent contact angle θ*=76.17° into Equation (6), the intrinsic contact angle is calculated as θ=81.17°, The predicted value θ was close to the tested value θ*. Thus, the idealized geometric model proposed in Figure 6 is effective, and it is valid to use the Wenzel wetting state model to calculate the intrinsic contact angle of the banana leaves’ adaxial side.

**Figure 7:**
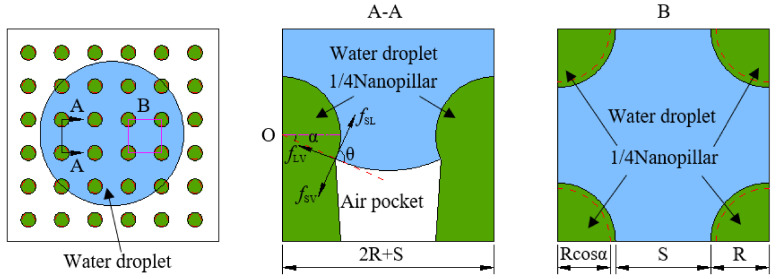
Idealized geometric model of the banana leaves’ abaxial side.

However, the weakly hydrophobic wetting state on the abaxial side of banana leaves was not suitable to be described by the Wenzel wetting state idealized geometric model. According to the Cassie–Baxter wetting state model (Figure 2c) and parameters of the nanopillars on the abaxial side of the banana leaves (Table 1), an idealized geometric model of a single nanopillar and the surrounding area is shown in Figure 7.

According to the Cassie–Baxter wetting state of the rough solid surface, the relationship between the apparent contact angle θ* and the intrinsic contact angle θ on the abaxial side of the banana leaf can be expressed as follows [23]:

(7) $$\cos\theta *=f_{SL}\cos\theta -f_{LV}$$

where $f_{SL}$ is the ratio of the solid–liquid interface to its projection on the abaxial side of the banana leaf and $f_{LV}$ is the ratio of the liquid–vapour interface to its projection on the abaxial side of the banana leaf. According to the idealized geometric model of the nanopillars and the water droplet in Figure 7, $f_{SL}$ and $f_{LV}$ can be expressed as:

(8) $$f_{SL}=\frac{2\pi R^{2}\left(1+\sin\alpha \right)}{{\left(S+2R\right)}^{2}}$$

(9) $$f_{LV}=\frac{{\left(S+2R\right)}^{2}-\pi {\left(R\cos\alpha \right)}^{2}}{{\left(S+2R\right)}^{2}}$$

where α is wetting angle, and α can be obtained by substituting the parameters of the nanopillars and the contact angle. Combining Equations (7) and (9), Equation (7) can be rewritten as:

(10) $$\cos\theta *=\frac{2\pi R^{2}\left(1+\sin\alpha \right)\cos\theta +\pi {\left(R\cos\alpha \right)}^{2}}{{\left(S+2R\right)}^{2}}-1$$

Similarly, the Cassie–Baxter wetting state idealized geometric model can be used to describe the weakly hydrophobic wetting state of the abaxial side of the banana leaves.

## 4. Conclusions

In this work, the wetting state of both the adaxial and abaxial sides of the banana leaves was studied. First, the surface of the fresh banana leaves was characterized by using advanced modern instrumental analysis techniques and methods. Second, the dynamic impact test was used to study the different dynamic responses of the banana leaf surface. Finally, an idealized geometric model of the banana leaf surface was established, revealing the underlying mechanism of the Janus wettability on the adaxial and abaxial sides of the banana leaves. The main results of this study are as follows:

1. Scanning electron microscopy studies on the banana leaf surface found that the nanopillars on the adaxial side were cone-shaped with large gaps, and the heads of the nanopillars on the abaxial side were smooth and spherical with small gaps. The contact angle test found that the banana leaf surface has high adhesion properties (RA > 90°), and the banana leaf exhibits a Janus wetting state where the adaxial side was hydrophilic (CA = 76.08° ± 2.01°) and the abaxial side was weakly hydrophobic (CA = 94.32° ± 1.04°). This is caused by the different morphology and distribution of the nanopillars on the adaxial and abaxial sides of the banana leaf.
2. A dynamic impact test was carried out to study the wettability of the banana leaf surface. The study found that when water droplets hit the adaxial and abaxial sides of the banana leaves, they showed different dynamic responses, and the wettability of the adaxial side was always stronger than the abaxial side. The change trend of the spreading factor under different impact velocities was the same, and with the increase in the impact velocity, when the impact velocity reached 1.4 m·s^−1^, the maximum spreading factor and the final steady-state spreading factor showed a small peak, achieving the best effect.
3. According to the information of the parameters and structure of the nanopillars on the banana leaf surface, an idealized geometric model of both the adaxial and abaxial sides of the banana leaf was established, and the classical wetting theory model was introduced to reveal the Janus wettability transition from the Wenzel state to Cassie–Baxter states of the banana leaf surface and the underlying mechanism.
  The study of Janus wettability on banana leaf surface can provide an important reference for the design and manufacture of bionic nanostructured surfaces with Janus wettability and the exploration of efficient and high-quality production management of banana orchards. In the future, on the basis of current research, we will use banana leaves as a biological template to prepare a series of surface materials with Janus wettability and conduct practical production and application research in the fields of droplet condensation and fog harvest.

## Figures and Tables

**Table 1:** Parameters of nanopillars on the banana leaf surface.

Parameters	Adaxial Side	Abaxial Side
R (nm)	66.75 ± 0.82	70.22 ± 1.80
H (nm)	364.19 ± 00.69	260.89 ± 1.00
S (nm)	201.83 ± 4.06	173.97 ± 0.58

**Note:** R is the radius of the nanopillars structure, H is the height, and S is the spacing. The parameters are composed of the average value and standard deviation of three repeated measurements.

## Data Availability

Not applicable.
